# Dance to be creative: the creative beliefs, creativity ability and improvisation performance in experts, amateurs, and non-dancers

**DOI:** 10.3389/fpsyg.2026.1670292

**Published:** 2026-07-02

**Authors:** Anahita Soltantouyeh, Tommaso Feraco, Benedetta Agostinis, Chiara Meneghetti

**Affiliations:** Department of General Psychology, University of Padova, Padua, Italy

**Keywords:** creative mindset, creative self-efficacy, creativity performance, improvisation task, modern dance, self-concept

## Abstract

**Introduction:**

The connection between dance and creativity offers a promising avenue for exploration. This study examines the relation between modern dance and creative performance (cognitive and motor) as well as creative self-beliefs.

**Methods:**

Expert dancers (35), amateur dancers (45) and non-dancers (controls, 36) performed a creative task and completed questionnaires measuring creative beliefs (self-concept, self-efficacy, and growth mindset). Experts and amateurs also performed a dance improvisation task.

**Results:**

(i) Experts performed better in both creative and improvisation tasks; amateurs showed higher creative performance than controls; (ii) experts (but not amateurs) reported higher creative self-beliefs than controls; (iii) improvisation performance was predicted by creative task scores in amateurs but not in experts, who likely rely more on their domain-specific expertise.

**Discussion:**

The results confirm links between dance and creative performance (cognitive and motor) and highlight the role of creative beliefs as a function of expertise.

## 1 Introduction

Creativity is increasingly recognized as a key skill for societal and personal success (World Economic Forum, 2020; OECD, 2021). In psychology, creativity is commonly defined as the ability to produce work that is both novel (original) and appropriate (useful and adaptive to task constraints) (Sternberg and Lubart, 1998).

Dance is a form of human expression grounded in bodily movement. Modern and contemporary dance emphasize free movement exploration and personal interpretation. Unlike routine physical activities, dance engages multiple cognitive and affective systems (including motor, emotional, imaginative, and neurocognitive processes) making it a real domain for studying creativity (Foster Vander Elst et al., 2023; Richard et al., 2024).

Embodied cognition provides a framework for understanding dance in relation to cognition. According to this framework cognitive processes are understood as deeply rooted in the body’s interactions with the world, relying on perceptual and motor systems rather than abstract symbol manipulation alone (Wilson, 2002). From this perspective movement-based activities such as dance are thought to engaging cognitive processes through action, including processes relevant to creative thinking. Building on this embodied view, the broader concept of “embodied creativity” refers to creative processes that are shaped by bodily experience and sensorimotor engagement (Malinin, 2019). Within this framework, the more specific notion of “kinaesthetic creativity” focuses on the body’s capacity to generate novel movement ideas (Hsueh et al., 2019). It is a domain-specific expression of embodied creativity, particularly relevant to dance, in which the moving body itself becomes the medium for creative ideation. This distinction allows the present study to examine in dance both cognitive creativity and motor creativity in improvisation.

### 1.1 Physical activity, dance and creative performance

Empirical evidence showed the relation between general physical activity and dance on cognitive tasks performance measuring creative ability. Creativity performance is typically assessed through divergent thinking tasks, such as the Alternative Uses Task (AUT; Guilford et al., 1960) and the Remote Associates Test (RAT; Mednick and Mednick, 1967), which measure fluency, flexibility, and originality of thought.

*Physical activity, dance and cognitive creative tasks*. Physical movement in space has been linked to creative performance (Kuo and Yeh, 2016; Leung et al., 2012; Malinin, 2019; Zhao et al., 2023). Leung et al. (2012) found that being situated inside or outside of a *cardboard* box (symbolizing thinking outside of the box) impacted the participants’ creative solutions (RAT). Kuo and Yeh (2016) showed that a group freely walking around - had higher creative performance (AUT) compared a group walking on a fixed line. Further Zhao et al. (2023) demonstrated that a group participating in constant moderate aerobic exercise showed significantly enhanced creative performance (fluency, RAT) compared to those exercising infrequently. These findings highlight that bodily movements and contexts can cue cognitive flexibility and novel perspectives.

Dance, as a particular subset of physical activity, is associated with creative performance in a unique way. In a study by Bollimbala et al. (2023), one group participated in a 15-min freestyle Bollywood dance session, while another group cognitively engaged in a topic discussion (control group). The results showed that although the two groups were similar in creative task performance before the intervention, the dance group achieved greater creative flexibility and originality on the divergent thinking task after the session (using AUT; Guilford et al., 1960). Moreover, dance appears to be associated with creativity through mechanisms not shared by other physical activities. Rehfeld et al. (2018) compared dancers to people engaging in equally intense fitness routines and found that only dancers showed lasting improvements in memory, attention, and a key brain-growth marker tied to learning, suggesting that dance involves neurocognitive processes beyond those associated with physical exertion alone.

Neuroimaging and electrophysiological research help clarify the neural basis of this advantage. Dance training produces structural and functional adaptations in motor control and sensorimotor networks, including premotor cortex, supplementary motor area, and basal ganglia (Burzynska et al., 2017; Hackney et al., 2024). Critically, these same networks, along with prefrontal and parietal regions, are also recruited during divergent thinking tasks (Cogdell-Brooke et al., 2020; Beaty et al., 2016). This network overlap provides a neurofunctional basis for the link between dance expertise and creative performance. Consistent with this, Ermutlu et al. (2015) showed that dancers exhibit distinct electroencephalographic patterns compared to fast-ball sport athletes, particularly increased alpha-band coherence in frontal and parietal regions. Fink et al. (2009) also found that professional dancers show stronger parietal alpha synchronization during creative ideation than novices, this showed positive correlation with superior divergent thinking scores in their study. In another experiment, the degree of functional brain adaptation in dancers was positively associated with scores on originality and flexibility measures (Yang et al., 2023). Additionally, Yu et al. (2025) found that dancers display specific patterns of cortical morphometric change in motor and sensorimotor areas that are partially shared with musicians but also show dance-specific features. Together, these findings suggest that dance practice engages neural networks shared with other sports and artistic activities, while also recruiting dance-specific neural networks involved in creative cognitive performance.

*Dance and motor creativity in improvisation.* Motor creativity actions often “emerge in the act” of moving rather than being pre-planned, as dancers discover novel solutions by exploring movement variability at the moment. This dynamic aligns with the concept of divergent thinking in action, where the dancer’s body generates multiple possibilities for movement, leading to innovations that might not surface through thought alone (Orth et al., 2017).

Movement generation in dance is related to creative cognitive ability (Torrents Martin et al., 2015; Richard et al., 2018, 2020, 2021, 2024). Torrents Martin et al. (2015) observed that when dance students were tasked with choreographing under movement constraints, they demonstrated improved creative problem-solving, suggesting that navigating physical constraints can sharpen divergent thinking. Richard et al. (2018) proposed a 10-session physical education program -including improvisation and movement awareness- for fourth-grade students and this group was compared with peers following a conventional syllabus (traditional class activity). At the end of the program, the intervention group showed greater motor fluency, flexibility, and originality in a movement invention task, as well as higher divergent thinking scores (Many Uses task using objects). Further, Richard et al. (2021) working with university students compared 5-week programs in improvisation, aerobic dance, and reading. Only the improvisation group improved significantly in both motor creativity (number and variety of movements) and cognitive creativity (using a Picture Completion task, while the other groups showed little change). Together, these findings demonstrate that improvisation of movement training consistently enhances motor and cognitive creativity.

In trained dancers, the picture becomes more complex. Fink and Woschnjak (2011) found that across styles, modern and contemporary dancers -whose daily practice often involves improvisation- outperformed ballet dancers on both verbal and figural creativity tasks, (assessed with Unusual Uses and Picture Completion tasks, respectively). Jazz/musical dancers generally fell between these two groups, though their differences were not statistically significant. Issartel et al. (2017) compared dancers of varying expertise levels as they improvised both alone and in pairs. Using kinematic analysis, they showed that experts produced more complex, organized, and varied movement patterns than novices, and that this expertise was evident even in socially coordinated (joint) improvisation. However, Palmiero et al. (2019) showed that even among young ballet dancers, domain-specific creative advantages emerge: dancers (6–10 years old) outperformed non-dancers only in a motor-divergent thinking task (by creating novel motor sequences) while they did not differ on a general divergent thinking task. This motor-creative edge was partly explained by a more precise topological body map, connecting bodily awareness developed through dance to motor-domain creativity. These findings suggest that sustained dance improvisation practice correlates significantly with creative performance.

In sum, the literature overview suggests that the performative nature of dance -especially the spontaneous invention of movements- offers a real-world context for practicing and refining creative cognitive skills such as ideation, adaptation, and innovation. Repeated engagement in improvisation has been linked to higher cognitive creative performance like fluency and originality, suggesting that these skills may transfer beyond movement into broader domains of creative thinking.

### 1.2 Dance and creative beliefs

Some evidence suggests that dance experience relates to beliefs about one’s creative identity. Creative beliefs are self-perceptions of creativity that shape and stabilize creative behavior over time encompassing creative self-efficacy (confidence in generating creative outcomes), creative identity (seeing oneself as creative), and growth mindset (the belief that creative abilities can be developed), and they are generally assessed through self-report questionnaires (Karwowski and Kaufman, 2017). These constructs are not merely reflections of ability; creative self-beliefs are theorized to shape and stabilize creative behavior over time, forming a reciprocal relationship with both creative performance and bodily engagement in expressive practice (Karwowski and Kaufman, 2017). In the context of dance, questions about how movement practices are related to creative beliefs -and become part of individuals’ personal schema- are particularly important.

Some evidence shows that dance practice is related to creative beliefs. A systematic review showed that dance interventions -particularly those with creative or expressive elements- tend to promote specific aspects of the self, such as self-expression and social self-concept, although effects on global self-esteem or body attitudes are mixed (Schwender et al., 2018). Richard et al. (2024) examined a 15-week creative intervention with university students (combining improvisation, expressive tasks and choreography), compared to a control group enrolled in a double degree course, to assess its impact on ideational behaviors, tolerance of ambiguity, emotional creativity, and creative self-efficacy. Specifically, students in the intervention condition improved self-reported ideational behaviors and remained stable in their tolerance to ambiguity compared to the control group, with descriptive gains in emotional creativity and creative self-efficacy. This suggests that immersive engagement in improvisational and expressive movement can reinforce not only creative skills but also individuals’ perception of their creative capabilities.

Qualitative evidence offers additional nuances. Braun and Kotera (2022) using interpretative phenomenological analysis, found that participants in a creative movement group viewed dance as a means of accessing and expressing their inner selves, enhancing their self-awareness and wellbeing. For many, improvisational movement served as a way to “tune into” their creative potential and gain confidence in self-expression (Aujla and Farrer, 2015). This sense of creative self-perception contributed to a stronger feeling of ownership and identity as artists, suggesting a connection between creative engagement and the development of self-concept.

In parallel, Mansour et al. (2018) examined the development of arts self-concept—i.e. how individuals perceive their abilities across creative fields, including dance—while creative self-efficacy specifically refers to confidence in producing creative outcomes. The study highlights a reciprocal relationship between arts self-concept and participation in creative and performing arts. Active engagement—such as dancing or taking arts lessons outside of school—was linked to higher self-concept over time, suggesting a virtuous cycle: participation boosts confidence in artistic abilities, which in turn encourages further involvement. While situated in a broader arts context, the study emphasizes that active practice, rather than mere exposure, is key to developing a creative self-concept. Survey findings also suggest that improvisational dance fosters traits linked to creative potential. Students with improvisation training scored higher on personality dimensions such as boldness, sensitivity, and imaginative thinking—traits that support creative risk-taking—compared to their untrained peers (Dou et al., 2021). This aligns with performance-based evidence, indicating that improvisation may help shape the very dispositions that support creative behavior (Fink and Woschnjak, 2011).

Overall, evidence in the literature suggests that dance practice involving improvisation is associated with creative cognitive performance (Fink and Woschnjak, 2011; Richard et al., 2018, 2021) with some evidence indicating that dancers perceive themselves as creative agents (Karwowski and Kaufman, 2017; Mansour et al., 2018). However, research on creative beliefs and how they relate to creative performance, whether in cognitive or improvisation tasks, remains limited and deserves to be further investigated. Therefore, the present study investigates how dance experience is related to creative performance, encompassing both creative cognitive tasks and creative movement, such as improvisation, as well as creative beliefs.

### 1.3 Rationale of the study, aims and hypotheses

The present study examines how expertise in contemporary dance relates to creative performance, both in cognitive tasks and in movement-based improvisation, as well as to creative beliefs. Prior work has shown that dance experience is associated with enhanced divergent thinking (Fink and Woschnjak, 2011; Palmiero et al., 2019), as well as higher performance on motor creativity—based on improvisation task (Richard et al., 2018, 2021)—and that these outcomes correlate with the degree of expertise (Issartel et al., 2017). Improvisation has been proposed as a key window into creative movement, suggesting that flexibility and variety of improvised actions reflect underlying creative thinking. Although still limited, emerging evidence suggests that creative beliefs—such as arts self-concept (Mansour et al., 2018), creative self-expression and embodied self-awareness (Braun and Kotera, 2022) and self-efficacy (Richard et al., 2024) may be shaped through creative dance practice. Yet the role of these beliefs in relation to creative performance, particularly in cognitive domains, remains underexplored. Building on this literature, the study examines how contemporary dance expertise relates to both creative performance (cognitive and motor) and creative beliefs. Specifically, the aim of the present study is threefold: (Aim 1) to examine the relationship between dance experience and creative performance (both cognitive and movement-based); (Aim 2) to examine the relation between dance experience and creative beliefs; and (Aim 3) to explore how dance experience, creative performance, and creative beliefs interrelate.

To address these aims, two groups of dancers, experts and amateurs, and a control group of non-dancers performed a cognitive creativity task (AUT), and completed questionnaires assessing creative beliefs, including creative identity, creative self-efficacy, and growth mindset. Growth mindset, defined as the belief that abilities can be improved (Yeager and Dweck, 2020), was included because it has been linked to performance across domains such as sports, academics, and the workplace (Brady and Alleyne, 2017; Feraco et al., 2023; Kondratowicz and Godlewska-Wererska, 2022). A fluid intelligence test was also administered, given the established link between fluid intelligence and creativity (Nusbaum and Silvia, 2011). Only the two dancer groups, experts and amateurs, completed the movement improvisation task.

Therefore, according to the aims, we outline the following expected results:

(i) Dance experience and creative performance (Aim 1). Dancers (experts and amateurs) are expected to perform better on cognitive tasks (AUT) compared to controls. Differences between experts and amateurs will be examined, with the possibility of detecting an advantage for experts. Regarding improvisation, expert dancers are expected to perform better than amateurs.
(ii) Dance experience and creative beliefs (Aim 2). Dancers (particularly experts) are expected to report stronger creative self-concept and higher self-efficacy compared to non-dancers. Furthermore, dancers might report higher levels of growth mindset in line with evidence from other domains. If these beliefs become integral to dancers’ self-concept, we anticipate experts to show more developed creative beliefs than amateurs.
(ii) Dance experience, creative performance, and beliefs (Aim 3). Cognitive creativity (e.g., divergent thinking), creative self-concept, self-efficacy, and growth mindset are expected to be positively related with improvisation performance. The improvisation task is of particular interest, as it may serve as an embodied expression of creativity—externalizing not only cognitive processes but also one’s creative self. In this way we predict that improvisational performance should be supported by creative cognitive performance, as well as dancer’s creative beliefs (self-concept, self-efficacy and growth mindset). Differences between amateurs and experts will also be examined.

## 2 Method

### 2.1 Participants

A total of 116 participants (88 females; M_age_ = 24.02; SD_age_ = 7.61) took part in this study, distributed across three groups (see Table 1). The expert group (*n* = 35; 27 females; M_age_ = 31.17; SD_age_ = 6.10) included individuals with extensive experience in contemporary dance, specifically at least 3 years in a professional dance academy and a minimum of 1 year working as professional dancers (year of experience, M = 17.81; SD = 8.17). The amateur group (*n* = 45; 43 females; M_age_ = 18.04; SD_age_ = 3.42) consisted of individuals who had practiced contemporary dance for at least 3 years but had no professional experience or formal academy training (year of experience, M = 9.84; SD = 5.13). The control group (*n* = 36; 18 females; M_age_ = 24.53; SD_age_ = 6.51) comprised non-dancers with no prior dance experience and no regular engagement in sports (defined as no more than 4 h per week of non-competitive physical activity).

**Table 1:** Descriptive statistics (M and SD) of the three groups (controls, amateurs and experts) and correlations of all variables (creative mindset, creative identity, creative self-efficacy, creative fluency, fluid reasoning) in the whole sample.

	Controls	Amateurs	Experts	Creative mindset	Creative identity	Creative self-efficacy	Creative fluency	Improvisation creativity
1. Creative mindset	3.58 (0.48)	3.78 (0.47)	3.89 (0.42)	1				
2. Creative identity	3.43 (0.97)	3.66 (0.84)	4.26 (0.55)	0.40***	1			
3. Creative self-efficacy	3.42 (0.68)	3.28 (0.66)	3.75 (0.52)	0.26**	0.63***	1		
4. Creative fluency	11.03 (4.50)	16.82 (7.92)	16.37 (7.61)	0.25**	0.25**	0.32**	1	
5. Fluid reasoning	8.97 (1.59)	10.24 (2.61)	8.91 (2.06)	0.08	−0.07	−0.07	0.24**	1
6. Improvisation creativity	—	2.88 (1.09)	4.29 (1.00)	−0.02	0.33**	0.35**	0.07	−0.24**

*p < 0.05; **p < 0.01; ***p < 0.001.

The 3 years as criterion of inclusion is due for practical reasons (availability of professional group) even if it is inconsistent in profiling dance as expert which is a combination of professional status, cumulative practice hours, and current training load as key criteria (Hutchinson et al., 2013; Kawalek and Gobet, 2022). Experts had significantly more years of dance experience than amateurs (*t*(70) = 4.56, *p* < 0.001), and were, on average, older (*t*(78) = 12.20, *p* < 0.001, M_age diff_ = 13.13). The ratio of females was significantly higher in the amateur group compared to both the control and expert groups (*t*(2) = 22.71, *p* < 0.001).

A posteriori power analysis indicated that detecting a medium-to-large effect size (*d* = 0.63) with 80% power would require approximately 40 participants per group. However, recruiting larger samples—particularly of elite dancers—was constrained by their limited availability, intense training schedules, and the rarity of individuals meeting the strict expertise criteria.

The study was approved by the Ethics Committee of the University of Padova (Protocol No. 4551). All participants, or their legal guardians if under 18, provided informed consent prior to participation.

### 2.2 Materials

*Short Scale of Creative Self* (Karwowski et al., 2018). This 11-item scale assesses two components of creative beliefs: creative self-efficacy (6 items; e.g., “I trust my creative abilities”) and creative identity (5 items; e.g., “I think I am a creative person”). Responses are given using a 1–5 Likert scale. Separate subscale scores are calculated. Both subscales are highly reliable, with Cronbach’s alpha values of 0.83 for creative identity and 0.84 for creative self-efficacy (Karwowski et al., 2018).

*Creative Mindset Scale* (Karwowski, 2014). This 10-item scale assesses beliefs about the malleability of creativity: 5 items reflect a growth mindset (e.g., “It doesn’t matter what creativity level one reveals—you can always increase it”), while 5 items reflect a fixed mindset (e.g., “You have to be born a creator—without innate talent you can only be a scribbler”). Responses are given on 1–5 Likert scale. Fixed mindset items are reverse-scored, and a total score is computed, with higher values indicating a stronger growth mindset. Cronbach’s alpha of the Creative Mindset Scale is acceptable (i.e., >0.70; Karwowski, 2014).

*Guilford Alternate Uses Test* (AUT; Guilford et al., 1960). This test assesses divergent thinking ability by asking participants to list as many alternative uses as possible for two common objects (a brick and a journal), with a time limit of 4 min per item. The total score is the sum of all valid alternative uses generated across both items (evaluated by a judge according AUT manual criteria). The validity and reliability of the AUT tests are generally very high (Saretzki et al., 2024).

*Dance Improvisation Task* (adapted from Clements et al., 2018). This task, designed for dancers, involves performing an improvised dance based on the following prompt: “The body is a clock: it has circadian or longer rhythms—sleep-wake, day-night, tides, seasonal shifts. The body is a rhythmic system, so it needs rest to continue, cycling between rest and activity” (Kirsh et al., 2020). Performances were recorded and anonymously evaluated by two expert judges (i.e., contemporary dancers, choreographers, or artistic directors of national dance companies). The Consensual Assessment Technique (Amabile, 1982) was used to assess the creativity of the improvisation performances across four dimensions—creativity, technique, pleasure, and meaning—using a 1–7 Likert scale. For this study, only the creativity rating was analyzed. Each participant’s creativity score was calculated as the average of the two judges’ ratings. The inter-rater reliability between judges was 0.83.

*Culture Fair Intelligence Test* (Cattell, 1940). This test assesses fluid reasoning ability through four timed subtests. For this study, we administered two: *matrix completion* (13 items, 3 min) in which participants identify the missing piece in a matrix by selecting the correct alternative; and *spatial relationships* (10 items, 2 min) in which participants identify spatial relationships between figures and select the correct alternative maintaining such relationships. One point was given for each correct answer, while incorrect or unanswered items received zero points. The split-half reliability of the test in the Italian population is 0.80 and its test–retest reliability 0.84 (Cattell, 1940).

### 2.3 Procedure

Participants were recruited through word-of-mouth. For dancers, several cultural associations and dance companies were also contacted via an introductory letter signed by the research coordinator. Expert dancers were additionally recruited through the third author’s direct connections with professional dance schools and academies. All participants voluntarily agreed to take part in the study and signed an informed consent form.

The procedure consisted of two sessions for dancers and one session for non-dancers. The first session, common to all participants, lasted approximately 30 min and was conducted in small groups via Zoom, using the Qualtrics platform. Researchers scheduled the meetings, shared the Qualtrics link, and guided participants through the entire process. During this session, participants completed a demographics form, performed the AUT, filled out the questionnaires, and finally performed the Culture-Fair Intelligence Test.

Dancers (both amateurs and experts) participated in a second individual session lasting about 10 min, held within 2 weeks of the first session. During this session, conducted via Zoom, participants were asked to perform a dance improvisation.

## 3 Results

All analyses were conducted using R (R Core Team, 2022). Descriptive statistics and intercorrelations among all variables are presented in Table 1. Standardized means and standard deviations for the three groups are also visualized in the Supplementary Figure S1.

### 3.1 Group differences in creative task, improvisation task and beliefs (aims i and ii)

*Statistical analyses*. Descriptive statistics indicate that the three groups differ consistently across the variables of interest (creative fluency, creative mindset, creative identity, creative self-efficacy, and improvisational creativity). To test these differences, we conducted five multiple linear regression models. Each model included sex (to account for gender imbalance across groups) and fluid reasoning (given its known relationship with creativity performance; Nusbaum and Silvia, 2011) as covariates, and group as the main predictor. The control group served as the baseline for all models except the improvisation performance, where amateurs were used as the reference group and compared with experts. Age was not included as a covariate due to its strong association with group membership (experts were older due to greater experience). Importantly, since age tends to negatively correlate with cognitive performance (e.g., fluency), the higher performance of older experts may indicate particularly robust creative abilities, reinforcing the observed group differences.

**Figure 1:**
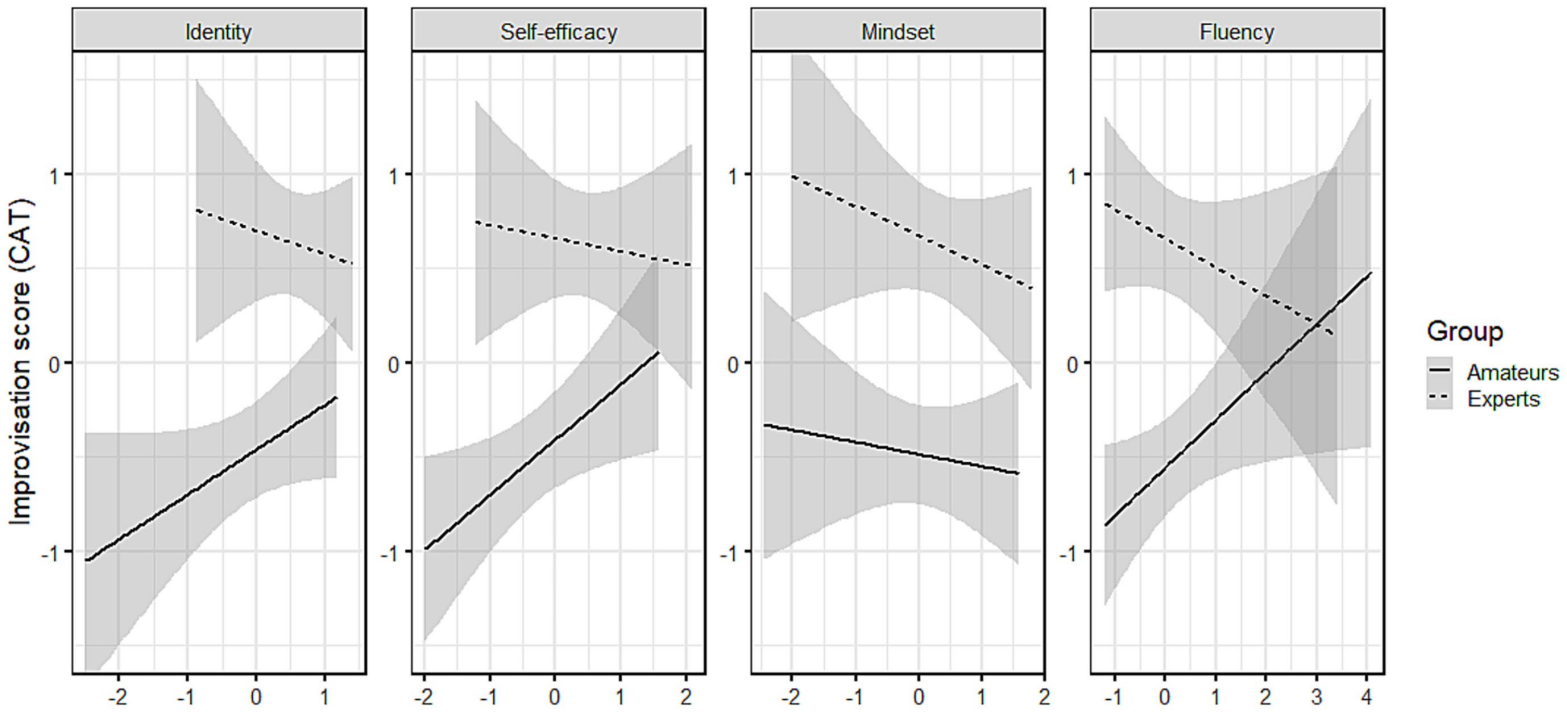
Effect of creativity indices (identity, mindset, self-efficacy, and fluency) and dancing expertise on CAT improvisation performance (significant interaction: Creative fluency × group).

Regression results (see Table 2) show that expert dancers significantly outperformed controls across all measures (*ps* < 0.05), with standardized beta coefficients ranging from 0.54 to 0.92 (see Figure 1). In improvisation, experts also significantly outperformed amateurs (*β* = 1.04, *p* < 0.001). Amateurs scored significantly higher than controls only on creative fluency (AUT; *β* = 0.71, *p* < 0.01). Fluid reasoning was a significant predictor only for creative fluency (*β* = 0.20, *p* < 0.05), while no significant effects emerged for sex.

**Table 2:** *β* estimates of the five linear regression models on dependent variables (creative fluency, creative mindset, creative identity, creative self-efficacy, and improvisational creativity) considering as independent variable sex, fluid reasoning and group.

	Creative fluency	Creative mindset	Creative identity	Creative self-efficacy	Improvisation performance
Intercept	−0.50	−0.35	−0.36	−0.10	−0.43
Sex	−0.03	−0.02	0.05	−0.04	−0.06
Fluid reasoning	0.20*	0.07	−0.04	−0.001	−0.09
Amateurs°	0.71**	0.38	0.22	−0.16	—
Experts°	0.75**	0.67**	0.92***	0.54*	1.04***
*R* ^2^	0.16	0.07	0.15	0.09	0.32

*p < 0.05; **p < 0.01; ***p < 0.001; °Control group as baseline.

### 3.2 Relation between creative task, improvisation task and beliefs (aim iii)

*Statistical analyses*. The correlation matrix (Table 1) based on the entire sample shows that self-reported beliefs about one’s creativity are positively associated with one’s actual ability in a creativity task. Additionally, creative self-efficacy and identity are also positively associated with the level of creativity in the improvisation performance.

Given the differences between experts and amateurs in creative (cognitive and improvisation) performance and beliefs, we decided to run a series of additional regression analyses to test whether these associations are similar across groups To this aim, we fit four linear regressions with improvisation performance as dependent variable and the interaction between each of the four creativity scores (the three scores of the two questionnaires and tsshe score of the alternative uses task) and dancers’ group (amateur vs. experts) as predictors.

Results (for details see in Supplementary Table S1) show a stable group difference (*p_s_* < 0.001) in favour of expert dancers in creative mindset (*β* = 1.16, *p* < 0.001), creative identity (*β* = 1.16, *p* <0.001), creative self-efficacy (*β* = 1.07, *p* <. 001) and creative fluency (*β* = 1.22, *p* < 0.001). Additionally, a significant interaction of creative fluency performance with groups emerged (*β* = −0.41, *p* < 0.05): among amateurs, creative ability significantly predicts improvisation performance, whereas in experts this relationship was not significant. A similar trend—even though the interactions were not significant—can be observed (see Figure 1) for creative identity (*β* = −0.36, *p* = 0.65) and creative self-efficacy (*β* = −0.36, *p* = 0.09).

## 4 Discussion of the results and conclusion

This study examined how expertise in contemporary dance relates to creative performance, both cognitive and movement-based improvisation, as well as creative beliefs. While dance has long been understood as an expressive art form, its role in creative thinking and personal identity has received growing empirical attention. Unlike structured physical activities, dance (especially modern/contemporary) inherently demands spontaneous movement generation, making it a natural model for studying creativity in action. We specifically examined whether dance expertise correlates with cognitive creative and improvisation performance (Aim 1), also if dance is related to creative beliefs, expanding the analyses to multiple constructs (self-identity, self-efficacy, and growth mindset) (Aim 2) as well as the relationship between improvisation performance and cognitive performance and beliefs across levels of dance expertise (Aim 3).

*Dance experience and creative performance (cognitive and motor creativity)*. Our results confirm and extend prior findings (e.g., Fink and Woschnjak, 2011; Richard et al., 2018, 2021) by showing that both dancer groups (experts and amateurs) outperformed non-dancers in creative performance, here examined using fluency scores from the AUT. Creative fluency was significantly higher among experts and amateurs relative to controls, with experts showing the largest effect. This reinforces the idea that dance experience is related to general divergent thinking skills.

Crucially, experts also significantly outperformed amateurs in improvisational performance, highlighting the added value of embodied expertise in real-time creative expression (Issartel et al., 2017). These findings support the notion that improvisation is not only a method for cultivating creativity but also a sensitive and ecologically valid metric of motor-based creative capacity. The fact that these effects emerged even though the expert group was significantly older than amateurs suggests that dance may buffer or even reverse age-related cognitive decline in creative domains. Notably, dance, although presenting similarities with, it also differs from other creative practices (e.g., music, visual art; Yu et al., 2025; Mansour et al., 2018) in the underlying neural mechanisms and in creative output. However, the involvement of body during improvisation makes the difference with other practices. This real-time integration of thinking and moving may account for the breadth of creative transfer observed here, spanning both cognitive and motor domains.

These performance advantages may be understood in light of neuroanatomical evidence. As outlined in the introduction, dance training reshapes motor and sensorimotor networks that overlap with those recruited during divergent thinking (Burzynska et al., 2017; Cogdell-Brooke et al., 2020; Beaty et al., 2016). Importantly, the extent of such neural reorganization has been shown to correlate with creativity scores in dancers (Yang et al., 2023). The graded advantage we observed from controls to amateurs to experts is in line with this pattern, and is consistent with the idea that greater dance experience is associated with neural networks supporting both motor and cognitive creativity.

The findings showing that dance experience is related to creative cognitive performance, and that experts performed better on the motor improvisation task, can be broadly contextualized within the framework of embodied cognition and, more specifically, “embodied creativity,” according to which movement and sensorimotor experience ground creative thinking (Hsueh et al., 2019).

*Dance experience and creative beliefs*. Our results showed that experts had higher self-evaluation in all constructs considered (identity, self-efficacy and mindset) and this is in line with previous evidence (Braun and Kotera, 2022; Mansour et al., 2018; Richard et al., 2024). Notably, only individuals with high dance experience (experts) showed stronger creative self-concept (identity) and creative self-efficacy than both amateurs and non-dancers. Innovatively, the experts showed a higher creative mindset compared to controls extending previous findings from other domains (Brady and Alleyne, 2017; Orvidas et al., 2018) to the context of dance. These results suggest that high levels of dance expertise are linked not just to skill acquisition, but to deeper shifts in self-perception. Importantly, amateurs did not differ significantly from controls on either belief measure—despite performing better than controls on the creative task —suggesting that beliefs may lag behind actual creative ability in early or moderate stages of expertise development.

*Dance experience, creative performance, and beliefs*. Creativity in the improvisation task, for both amateurs and experts were predicted by creative performance (fluency) and creative beliefs (creative identity, self-efficacy, growth mindset), even though the scores were higher in experts compared to amateurs. Interestingly, the interaction between group and creative task showed that among amateurs the improvisation performance was significantly predicted by creative fluency performance, a relationship not observed among experts. This mismatch may indicate that early-stage dancers rely more heavily on cognitive creative skills to perform improvisation tasks, whereas experts may draw more on internalized creative believes and expertise making it an expression of their creative identity. Experts appear consolidated in both their creative performance and beliefs aligning with models in creativity research (e.g., Karwowski and Kaufman, 2017) that propose self-perceptions not only reflect but also shape and stabilize creative behavior over time. The role of creative beliefs in improvisation is also in line with Torrents and Calvo-Estelrich (2026), who found that in middle-aged non-dancers, motor dance creativity was modestly associated with figural creativity and creative self-efficacy but not with other creativity measures, and that self-efficacy was the only significant predictor of improvisation performance in their regression models.

Taken together, our results show that modern/contemporary dance expertise relates both to greater cognitive creative performance (via fluency and improvisation tasks) and to greater creative beliefs encompassing self-identity, self-efficacy and growth mindset. A novel contribution of this study is the finding that increasing levels of expertise are associated with broader creative beliefs. Despite previous encouraging evidence (Braun and Kotera, 2022; Mansour et al., 2018; Richard et al., 2024) these results support that dance expertise is related to the creative self, including its multicomponential aspects. Furthermore, both creative cognitive abilities and beliefs appear essential for improvisation tasks, with cognitive creativity being more influential for less experienced dancers while expertise probably play a greater role among experts.

Despite the value of these findings, some limitations should be noted. First, the improvisation task was only administered to dancers (experts and amateurs), excluding the control group. While this decision was methodologically justified, it precluded a full-group comparison in an improvisation task and limits the generalizability of conclusions regarding embodied creativity. Second, our improvisation task required movements to be performed alone and not in a group. Given that the modern/contemporary dance is frequently practiced in group settings, and relational dynamics are linked to creative performance (Leach and Stevens, 2020) further studies should examine improvisation tasks performed both individually and in groups. Third, the role of age and gender merits a reflection. The amateur group included a disproportionately high number of females compared to the other groups. Although sex was included as a covariate and did not emerge as a significant predictor in any model, potential gender-related differences in creative self-beliefs or expressive engagement cannot be entirely ruled out, and future studies should aim for more balanced group compositions. Furthermore, the expert dancers were in average 13 years older respect to the amateurs’ dancers. Even if the increase of expertise is intrinsically related to age, dance expertise still remained related to cognitive functioning (Powell et al., 2026). Future studies should better take into account the age in dance group composition. Therefore, both gender and age should be better matched as a function of dance experience level in future studies. Finally, a limitation is related to the quasi-experimental design, which restricts our ability to draw causal conclusions about the relationships between factors. Only further longitudinal studies will allow for a better examination of whether movement (as improvisation movement) supports the development of enhanced creative skills and beliefs.

Our results have potential implications for promoting creative activities. The fact that even amateur-level dance experience was associated with enhanced creative fluency, without requiring professional commitment, suggests that structured improvisation sessions could serve as accessible interventions for boosting divergent thinking in educational settings (Richard et al., 2018, 2021). Furthermore, the fact that creative beliefs are consolidated only at the expert level suggests that programs aiming to build creative self-efficacy should not rely on skill development alone but should also incorporate reflective and metacognitive elements to help participants internalize their developing creative capacities. Over time, such approaches may enable individuals who do not initially perceive themselves as “creative” to cultivate greater creative self-confidence through dance (McKay et al., 2024). This is particularly relevant given that creative self-efficacy has been positively associated with adaptive coping, wellbeing, and the capacity to respond flexibly to stressful situations beyond artistic contexts (Orkibi, 2021), suggesting that the benefits of fostering creative beliefs through dance may extend into everyday life.

To conclude, although more evidence is needed, the current study suggests that dance is a unique activity related to creativity in both thought and identity. These findings support a growing recognition that movement-based arts are not peripheral but central to understanding and fostering human creative potential.

## Data Availability

The datasets presented in this study can be found in online repositories. The names of the repository/repositories and accession number(s) can be found at: doi: https://doi.org/10.6084/m9.figshare.29477996.

## References

[ref1] Amabile T. M. (1982). Social psychology of creativity: a consensual assessment technique. J. Pers. Soc. Psychol. 43, 997–1013. doi: 10.1037/0022-3514.43.5.997

[ref2] Aujla I. Farrer R. (2015). The role of psychological factors in the career of the independent dancer. Front. Psychol. 6:1688. doi: 10.3389/fpsyg.2015.01688, 26579059 PMC4626556

[ref3] Beaty R. Benedek M. Silvia P. Schacter D. (2016). Creative cognition and brain network dynamics. Trends Cogn. Sci. 20, 87–95. doi: 10.1016/j.tics.2015.10.004, 26553223 PMC4724474

[ref4] Bollimbala A. James P. S. Ganguli S. (2023). The impact of physical activity intervention on creativity: role of flexibility vs persistence pathways. Think. Skills Creat. 49:101313. doi: 10.1016/j.tsc.2023.101313, 38826717

[ref5] Brady A. Alleyne R. (2017). “Resilience and growth mindset in sport and physical activity,” in Positive Psychology in Sport and Physical Activity, eds. Brady A. Grenville-Cleave B. (Abingdon: Routledge).

[ref6] Braun N. Kotera Y. (2022). Influence of dance on embodied self-awareness and well-being: an interpretative phenomenological exploration. J. Creat. Ment. Health 17, 469–484. doi: 10.1080/15401383.2021.1924910

[ref7] Burzynska A. Finc K. Taylor B. Knecht A. Kramer A. (2017). The dancing brain: structural and functional signatures of expert dance training. Front. Hum. Neurosci. 11:566. doi: 10.3389/fnhum.2017.00566, 29230170 PMC5711858

[ref8] Cattell R.B. (1940) Culture Fair Intelligence Test (CFIT) [Database record]. APA PsycTests. doi: 10.1037/t14354-000

[ref9] Clements L. Redding E. Sell N. L. May J. (2018). Expertise in evaluating choreographic creativity: an online variation of the consensual assessment technique. Front. Psychol. 9:1448. doi: 10.3389/fpsyg.2018.01448, 30197611 PMC6117233

[ref10] Cogdell-Brooke L. Sowden P. Violante I. Thompson H. (2020). A meta-analysis of functional magnetic resonance imaging studies of divergent thinking using activation likelihood estimation. Hum. Brain Mapp. 41, 5057–5077. doi: 10.1002/hbm.25170, 32845058 PMC7643395

[ref11] Dou X. Jia L. Ge J. (2021). Improvisational dance-based psychological training of college students’ dance improvement. Front. Psychol. 12:663223. doi: 10.3389/FPSYG.2021.663223, 34122253 PMC8189290

[ref12] Ermutlu N. Yücesir I. Eskikurt G. Temel T. İşoğlu-Alkaç Ü. (2015). Brain electrical activities of dancers and fast ball sports athletes are different. Cogn. Neurodyn. 9, 257–263. doi: 10.1007/s11571-014-9320-2, 25834650 PMC4378580

[ref13] Feraco T. Casali N. Meneghetti C. (2023). Adaptability and grit: foundations for their joint contribution to students’ academic and nonacademic outcomes. Mind Brain Educ. 17, 175–184. doi: 10.1111/mbe.12367

[ref14] Fink A. Graif B. Neubauer A. (2009). Brain correlates underlying creative thinking: EEG alpha activity in professional vs. novice dancers. NeuroImage 46, 854–862. doi: 10.1016/j.neuroimage.2009.02.036, 19269335

[ref15] Fink A. Woschnjak S. (2011). Creativity and personality in professional dancers. Pers. Individ. Dif. 51, 754–758. doi: 10.1016/j.paid.2011.06.024

[ref16] Foster Vander Elst O. Foster N. H. D. Vuust P. Keller P. E. Kringelbach M. L. (2023). The neuroscience of dance: a conceptual framework and systematic review. Neurosci. Biobehav. Rev. 150:105197. doi: 10.1016/j.neubiorev.2023.105197, 37100162

[ref17] Guilford J. P. Christensen P. R. Merrifield P. R. Wilson R.C. (1960) Alternate Uses (ALTUS) [Database record]. APA PsycTests. doi: 10.1037/t06443-000

[ref18] Hackney M. Burzynska A. Ting L. (2024). The cognitive neuroscience and neurocognitive rehabilitation of dance. BMC Neurosci. 25:8. doi: 10.1186/s12868-024-00906-8, 39506634 PMC11539675

[ref19] Hsueh S. Alaoui S.F. Mackay W.E. (2019) “Understanding kinaesthetic creativity in dance”, In: Proceedings of the 2019 CHI Conference on Human Factors in Computing Systems Glasgow

[ref20] Hutchinson C. Sachs-Ericsson N. Ericsson A. (2013). Generalizable aspects of the development of expertise in ballet across countries and cultures: a perspective from the expert-performance approach. High Abil. Stud. 24, 21–47. doi: 10.1080/13598139.2013.780966

[ref21] Issartel J. Gueugnon M. Marin L. (2017). Understanding the impact of expertise in joint and solo-improvisation. Front. Psychol. 8:1078. doi: 10.3389/fpsyg.2017.01078, 28713301 PMC5492827

[ref22] Karwowski M. (2014). Creative mindsets: measurement, correlates, consequences. Psychol. Aesthet. Creat. Arts 8, 62–70. doi: 10.1037/a0034898

[ref23] eds. Karwowski M. Kaufman J. C. (2017). The Creative Self: Effect of Beliefs, Self-Efficacy, Mindset, and Identity. Amsterdam: Elsevier Academic Press.

[ref24] Karwowski M. Lebuda I. Wiśniewska E. (2018). Measuring creative self-efficacy and creative personal identity. Int. J. Creat. Problem Solving 28, 45–57. Available online at: https://scholar.google.com/citations?view_op=view_citation&hl=en&user=Pn3vpgQAAAAJ&cstart=100&pagesize=100&sortby=pubdate&citation_for_view=Pn3vpgQAAAAJ:v6PuF9mNY3oC

[ref25] Kawalek P. Gobet F. (2022). Expertise in contemporary dance: the roles of cognition, talent, and deliberate practice. J. Dance Educ. 24, 21–34. doi: 10.1080/15290824.2021.1988089, 37339054

[ref26] Kirsh D. Stevens C. J. Piepers D. W. (2020). Time course of creativity in dance. Front. Psychol. 11:518248. doi: 10.3389/fpsyg.2020.518248, 33384634 PMC7770173

[ref27] Kondratowicz B. Godlewska-Wererska D. (2022). Growth mindset and life and job satisfaction: the mediatory role of stress and self-efficacy. Health Psychol. Rep. 11, 98–107. doi: 10.5114/hpr/152158, 38084317 PMC10670786

[ref28] Kuo C. Y. Yeh Y. Y. (2016). Sensorimotor-conceptual integration in free walking enhances divergent thinking for young and older adults. Front. Psychol. 7:1580. doi: 10.3389/fpsyg.2016.01580, 27790178 PMC5061809

[ref29] Leach J. Stevens C. J. (2020). Relational creativity and improvisation in contemporary dance. Interdiscip. Sci. Rev. 45, 95–116. doi: 10.1080/03080188.2020.1712541

[ref30] Leung A. K. Y. Kim S. Polman E. Ong L. S. Qiu L. Goncalo J. A. . (2012). Embodied metaphors and creative “acts”. Psychol. Sci. 23, 502–509. doi: 10.1177/0956797611429801, 22477105

[ref31] Malinin L. H. (2019). How radical is embodied creativity? Implications of 4E approaches for creativity research and teaching. Front. Psychol. 10:2372. doi: 10.3389/fpsyg.2019.02372, 31695653 PMC6818493

[ref32] Mansour M. Martin A. J. Anderson M. Gibson R. Liem G. A. D. Sudmalis D. (2018). Young people’s creative and performing arts participation and arts self-concept: a longitudinal study of reciprocal effects. J. Creat. Behav. 52, 240–255. doi: 10.1002/jocb.146

[ref33] McKay A. S. Reiter-Palmon R. Coombes S. M. T. Coombs J. E. (2024). A meta-analysis of creativity training in organizational settings. Creat. Innov. Manag. 33, 587–602. doi: 10.1111/caim.12605

[ref34] Mednick S. A. Mednick M. T. (1967). Remote Associates Test, College, Adult, Form 1 and Examiner’s Manual. Boston, MA: Houghton Mifflin Company.

[ref35] Nusbaum E. C. Silvia P. J. (2011). Are intelligence and creativity really so different?: fluid intelligence, executive processes, and strategy use in divergent thinking. Intelligence 39, 36–45. doi: 10.1016/j.intell.2010.11.002

[ref36] OECD. (2021). OECD Economic Outlook. Available online at: https://www.oecd.org/en/publications/oecd-economic-outlook/volume-2021/issue-1_edfbca02-en.html (Accessed May 31, 2021)

[ref37] Orkibi H. (2021). Creative adaptability: conceptual framework, measurement, and outcomes in times of crisis. Front. Psychol. 11:588172. doi: 10.3389/fpsyg.2020.588172, 33510671 PMC7835130

[ref38] Orth D. van der Kamp G. J. P. Memmert D. Savelsbergh G. J. P. (2017). Creative motor actions as emerging from movement variability. Front. Psychol. 8:1903. doi: 10.3389/fpsyg.2017.01903, 29163284 PMC5671646

[ref39] Orvidas K. Burnette J. L. Russell V. M. (2018). Mindsets applied to fitness: growth beliefs predict exercise efficacy, value and frequency. Psychol. Sport Exerc. 36, 156–161. doi: 10.1016/j.psychsport.2018.02.006

[ref40] Palmiero M. Giulianella L. Guariglia P. Boccia M. D’Amico S. Piccardi L. (2019). The dancers’ visuospatial body map explains their enhanced divergence in the production of motor forms: evidence in the early development. Front. Psychol. 10:768. doi: 10.3389/fpsyg.2019.00768, 31024403 PMC6467967

[ref41] Powell M. Davidson J. W. Forde Thompson W. (2026). Understanding the cognitive and psychosocial implications of dance expertise in older adults: a scoping review. Empir. Stud. Arts. doi: 10.1177/02762374261424469

[ref42] R Core Team (2022). R: A Language and Environment for Statistical Computing. Vienna: R Foundation for Statistical Computing.

[ref43] Rehfeld K. Lüders A. Hökelmann A. Lessmann V. Kaufmann J. Brigadski T. . (2018). Dance training is superior to repetitive physical exercise in inducing brain plasticity in the elderly. PLoS One 13:e0196636. doi: 10.1371/journal.pone.0196636, 29995884 PMC6040685

[ref44] Richard V. Ben-Zaken S. Siekańska M. Tenenbaum G. (2020). Effects of movement improvisation and aerobic dancing on motor creativity and divergent thinking. J. Creative Behav. 55, 255–267. doi: 10.1002/jocb.450

[ref45] Richard V. Brown D. M. Y. Garcías S. Almarcha M. Cairney J. Torrents C. (2024). The exploration of the holistic and complex impacts of creative dance on creative potential enhancement. J. Creat. Behav. 58, 511–529. doi: 10.1002/jocb.673

[ref46] Richard V. Holder D. Cairney J. (2021). Creativity in motion: examining the creative potential system and enriched movement activities as a way to ignite it. Front. Psychol. 12:690710. doi: 10.3389/fpsyg.2021.690710, 34659006 PMC8514639

[ref47] Richard V. Lebeau J. C. Becker F. Boiangin N. Tenenbaum G. (2018). Developing cognitive and motor creativity in children through an exercise program using nonlinear pedagogy principles. Creat. Res. J. 30, 391–401. doi: 10.1080/10400419.2018.1530913

[ref48] Saretzki J. Forthmann B. Benedek M. (2024). A systematic quantitative review of divergent thinking assessments. Psychol. Aesthet. Creat. Arts 20, 1–20. doi: 10.1037/aca0000691

[ref49] Schwender T. M. Spengler S. Oedl C. Mess F. (2018). Effects of dance interventions on aspects of the participants’ self: a systematic review. Front. Psychol. 9:1130. doi: 10.3389/fpsyg.2018.01130, 30065676 PMC6056677

[ref50] Sternberg R. J. Lubart T. I. (1998). “The concept of creativity: prospects and paradigms,” in Handbook of Creativity, ed. Sternberg R. J. (Cambridge: Cambridge University Press).

[ref51] Torrents C. Calvo-Estelrich C. (2026). Motor dance creativity shows limited associations with other creative behaviours. Exploring domain-specificity in middle-aged adults. Hum. Mov. Sci. 106:103450. doi: 10.1016/j.humov.2026.103450, 41570407

[ref52] Torrents Martin C. Ric Á. Hristovski R. (2015). Creativity and emergence of specific dance movements using instructional constraints. Psychol. Aesthet. Creat. Arts 9, 65–74. doi: 10.1037/a0038706

[ref53] Wilson M. (2002). Six views of embodied cognition. Psychon. Bull. Rev. 9, 625–636. doi: 10.3758/BF03196322, 12613670

[ref54] World Economic Forum (2020) The future of jobs report 2020. Available online at: https://www.weforum.org/publications/the-future-of-jobs-report-2020/ (Accessed May 31, 2021)

[ref55] Yang C. Yu H. Hong T. Shih C. Yeh T. Chen L. . (2023). Trait representation of embodied cognition in dancers pivoting on the extended mirror neuron system: a resting-state fMRI study. Front. Hum. Neurosci. 17:1173993. doi: 10.3389/fnhum.2023.1173993, 37492559 PMC10364845

[ref56] Yeager D. S. Dweck C. S. (2020). What can be learned from growth mindset controversies? Am. Psychol. 75, 1269–1284. doi: 10.1037/amp0000794, 33382294 PMC8299535

[ref57] Yu Y. He H. Yang R. Yang L. Liu Y. Yao D. . (2025). Shared and distinct patterns of cortical morphometric inverse divergence and their association with empathy in dancers and musicians. Sci. Rep. 15:28572. doi: 10.1038/s41598-025-13416-2, 40764652 PMC12325637

[ref58] Zhao Y. Qin C. Liu D. (2023). Effects of 12 min aerobic exercise on creativity. PsyCh J. 12, 470–472. doi: 10.1002/pchj.647, 37188353

