# Supplementary material for: Dance to be creative: the creative beliefs, creativity ability and improvisation performance in experts, amateurs, and non-dancers
Source: Front Psychol. 2026 Jul 2;17:1670292. doi: 10.3389/fpsyg.2026.1670292 (PMC13372592; doi:10.3389/fpsyg.2026.1670292)
Supplement: Supplementary file 1 [file Table_1.DOCX]

# Supplementary Tables and Figures

Figure S1. Mean and standard deviations of all variables of interest in z scores for the three groups.


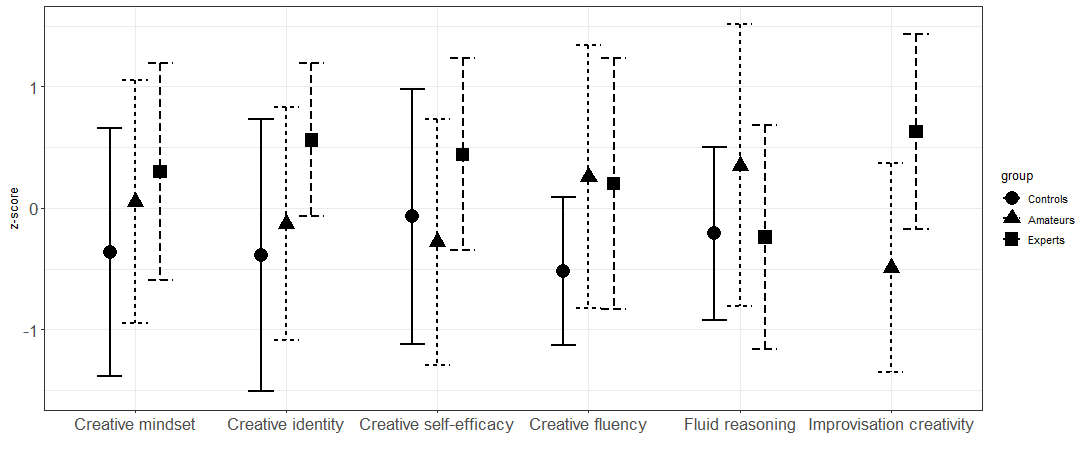


Table S1. Results (β) of the second set of regression analyses with improvisation performance as dependent variable.

| Predictors | Creative fluency | Creative mindset | Creative identity | Creative self-efficacy |
| --- | --- | --- | --- | --- |
| Intercept | -.56 | -.49 | -.46 | -.41 |
| Predictor effect | .25* | -.06 | .24 | .29* |
| Group effect (Experts vs Amateurs) | 1.22*** | 1.16*** | 1.16*** | 1.07*** |
| Interaction (group X predictor) | -.41* | -.09 | -.36 | -.36 |
| R^2^ | .36 | .32 | .34 | .36 |

* p <. 05, *** p <. 001, Experts > Amateurs
